# Biological structural study of emerging shaped nanoparticles for the blood flow in diverging tapered stenosed arteries to see their application in drug delivery

**DOI:** 10.1038/s41598-024-51848-4

**Published:** 2024-01-17

**Authors:** Noreen Sher Akbar, M. Bilal Habib, Maimona Rafiq, Taseer Muhammad, Metib Alghamdi

**Affiliations:** 1 DBS&H, CEME, National University of Sciences and Technology, Islamabad 44000, Pakistan; 2 COMSATS University, Islamabad, 44000 Pakistan; 3 Department of Mathematics, COMSATS University Islamabad, Attock, 43600 Pakistan; 4 Department of Mathematics, College of Science, King Khalid University, 61413 Abha, Saudi Arabia

**Keywords:** Biological techniques, Mathematics and computing, Nanoscience and technology

## Abstract

The magnetic force effects and differently shaped nano-particles in diverging tapering arteries having stenoses are being studied in current research via blood flow model. There hasn’t been any research done on using metallic nanoparticles of different shapes with water as the base fluid. A radially symmetric but axially non-symmetric stenosis is used to depict the blood flow. Another significant aspect of our research is the study of symmetrical distribution of wall shearing stresses in connection with resistive impedance, as well as the rise of these quantities with the progression of stenosis. Shaping nanoparticles in accordance with the understanding of blood flow in arteries offers numerous possibilities for improving drug delivery, targeted therapies, and diagnostic imaging in the context of cardiovascular and other vascular-related diseases. Exact solutions for different flow quantities namely velocity, temperature, resistance impedance, boundary shear stress, and shearing stress at the stenosis throat, have been assessed. For various parameters of relevance for Cu-water, the graphical results of several types of tapered arteries (i.e. diverging tapering) have been explored.

## Introduction

Rheology of streaming blood in human arteries has received a lot of attention recently and is a very exciting area of study for hemologists. Peristaltic pumping is the process by which the human body's cells are supplied with nutrients through oxygen-rich blood under a suitable pressure caused by a progressive wave of regular cardiac contraction and expansion, and subsequently blood or (bodily fluid) is returned together with cellular waste. However, because it depends on the hydrodynamic behaviour and mechanical characteristics of blood vessel walls, blood transport is not always as regular. Atherosclerosis, also known as stenosis in medicine, is a common disease caused by the narrowing of an artery's lumen as a result of the deposition of arteriosclerotic plaque or other types of abnormal tissues along the blood vessel wall. It may be the cause of the development of many cardiovascular diseases, particularly atherosclerosis (“athero” stands for “gruel or paste” and “sclerosis” defines “hardness”). By lowering or obstructing blood flow, this causes circulatory system disturbance, which may lead to the development of cardiovascular disorders such as heart attacks, hypertension, hypotension, stroke, etc. When the blood supply to a tissue is reduced or blocked, necrosis may eventually disappear. Therefore, in the current environment, it is crucial for scientific research to analyse blood flow in stenosed arteries^1^. Misra and Shit^2^, presented a numerical model describing effect of magnetic field on blood flow through an artery. In another article, a theoretical analysis for pulsating flow of blood, via porous conduit with externally applied magnetic field for an incompressible Newtonian fluid model flow is examined by Shit and Roy^3^. According to them, least velocity is observed at the throat of stenosis with maximum at the onset as well as outset of the stenosis. The micropolar fluid, to model blood flow via a tapering artery with a stenosis, was explored by Mekheimer and Kot^4^. They talked about the non-tapered, non-converging, and converging arteries. Tripathi^5^ discusses a mathematical model for the passage of food boluses with various viscosities via the oesophagus. Through Refs.^6–11^, additional recent literature can be viewed.

Nano fluid is currently a significant issue among researchers for a variety of reasons. The term "nanofluid" refers to a fluid rescheduling that includes ultrafine units having a span less than 50 nm, and Choi^12^ was the pioneer who popularised it. Brownian motion, nanoparticle bunching, and fluid layering at the liquid/solid boundary have all been implicated in the rise in thermal conductivity of nanofluids. Hamilton and Crosser^13^ presented the thermal conductivity of a heterogeneous two-component system. Recently Krishna et al.^14^ explored unsteady magnetohydrodynamic flow of nanofluid past porous plate placed vertically. They study roam about the behaviour of 2-different water based nanofluids. The results of their study reveals that an increase in the volume fraction of nanoparticles causes the temperature on the flow's central line to rise. Akram and Akbar^15^ investigated the fluid flow properties and heat transmission by the drilling muds augmented with nanoparticles flowing through the drilling pipes under different physical conditions theoretically. Consideration is being given to an essential kind of drilling fluid called Aphron, which is an excellent choice for drilling in depleted regions. The findings of their study show that when an advancing electric field is applied, the velocity profile radically surges and the temperature profile significantly declines. The mathematical modelling of water-based silver nanofluid propelled through peristaltic waves across an asymmetric conduit is the subject of research considered by Javeria et al.^16^. The mathematical modelling of the flow problem also takes into account the Joule heating phenomenon in addition to electroosmotic flow. Their study unveils the fact that backward electroosmosis inhibits peristaltic pumping while forward electric field promotes it, and the magnitude of the Nusselt number tends to increase as the Joule heating parameter increases. Iqbal et al.^17^ in their study demonstrates how mass and heat transfer affect the Powell-Eyring nanofluid's magnetohydrodynamic (MHD) bioconvective peristaltic transport across a curved channel with a radius-dependent magnetic field. The outcomes of the study show that a rise in curvature and a decrease in the Hartman number lower the axial velocity of nanofluid. Additionally, the concentration distribution gets better as the Brownian motion coefficient is increased. Nanoparticles with shape i.e. bricks cylinders and platelets etc. are also important. This phenomenon has been discussed by very few authors in literature. Ellahi et al.^18^ studied the shape effects of nanosize particles with entropy generation. For peristaltic flow of Cu-water nanofluid for various forms of nanosize particles, Akbar et al.^19^ discussed the peristaltic flow in tube with nanofluid. They take into account platelets, bricks, and cylinders as three different shape particle forms. Through the references, one can view current literature on the subject^20–24^. The conventional nanoparticles are very efficient in tumor targeting^25^, targeted supply of medication to tumor cells via nanoparticles, play an efficient role in chemotherapy via reverse of multiple drug resistance. The targeted nanoparticle delivery^26^ has efficient results in chemotherapy, molecular imaging, gene therapy. For chemotherapy of primary and advanced metastatic tumours, nanoparticles can transport medicines directly to cancer cells at a sustained rate that has the potential for greater effectiveness and fewer side effects^27^. The invention of nanotechnology drugs as a consequence of the rapid advancement of nanotechnology holds enormous potential to boost cancer treatment methods. The potential for versatility and novel targeting tactics in nanomedicine products emerges. They have been evaluated with multiple clinical utilizes, such as contrast agents in imaging, tumor-targeting gene delivery systems, and drugs carriers. For the development and advancement of innovative cancer therapies, various kinds of nanomaterials based on organic, lipid, inorganic, or glycan, as well as on synthetic polymers, have been used^28^. Some recent researches on the relevant topic are as follows^29–32^. In the present article, we have used copper nanoparticles. Kang et al.^33^ analyzed in their work how copper nanoparticles are being used as a promising anti-cancer agent. Aishajiang et al.^34^ investigated that copper may influence cancer cell viability by reactive oxygen species excessive buildup, and anti-angiogenesis, proteasome inhibition because tumor tissue has greater needs for copper and is more vulnerable to copper regulation. Because versatile copper-based nanotubes can be employed for both the detection and therapy of cancer, intracellular copper has been receiving a lot of interest. Further recent literature related to the topic can be found in Refs.^35–42^.

Blood can behave as both Newtonian and non-Newtonian fluid, it depends on the size of the arterial vessels. Blood acts as a Newtonian fluid inside the large arterial vessels where the shear rate has a value higher than 100 s^−1^. However, blood acts as a non-Newtonian fluid in small capillaries where the strain rate has low values. In our theoretical model, we have considered blood as a Newtonian fluid by considering water as a base fluid. We have considered the present model for a diverging tapered artery that is assumed to have a diameter of more than 100 μm that forms a large arterial vessel with shear stresses more than 100 s^−1^. Thus blood is treated as a Newtonian fluid for this theoretical study case by considering water as base fluid. The blood flow model is reused in light of the aforementioned analysis to investigate the effects of magnetic fields and variously shaped nanoparticles diverging tapering arteries.

The use of metallic nanoparticles in various shapes with water as the base fluid in connection with stenosis has not yet been investigated. Shaped nanoparticles can be engineered to interact with blood components in a way that prevents or mitigates the formation of blood clots (thrombosis). Shaped nanoparticles can serve as contrast agents for imaging techniques, offering improved visualization of blood vessels and blood flow patterns. This can aid in the early detection and diagnosis of vascular abnormalities. Certain shaped nanoparticles can be designed to respond to changes in blood flow or the local microenvironment. This responsiveness can be harnessed for controlled drug release, activated specifically in areas with altered blood flow patterns associated with disease states. Further certain nanoparticle shapes may reduce clearance rates and improve circulation times in the bloodstream. This is important for sustained drug release and prolonged exposure to the therapeutic agents and understanding blood flow dynamics aids in optimizing these properties. So the topic is very important from biomedical point of view. A radially symmetric but axially non-symmetric stenosis is used to depict the blood flow. One additional aspect of implementing current approach is the consideration of symmetry distribution for wall shearing stress in connection with resistive impedance, as well as the increase of these variables with the progression of stenosis. For velocity, resistance impedance, wall shear stress, and shearing stress at the stenosis throat, exact solutions have been assessed. The graphical results of different type of tapered arteries (i.e. diverging tapering) are analysed for multiple parameters involved in problem formulation.

## Mathematical formulation

**Figure 1:**
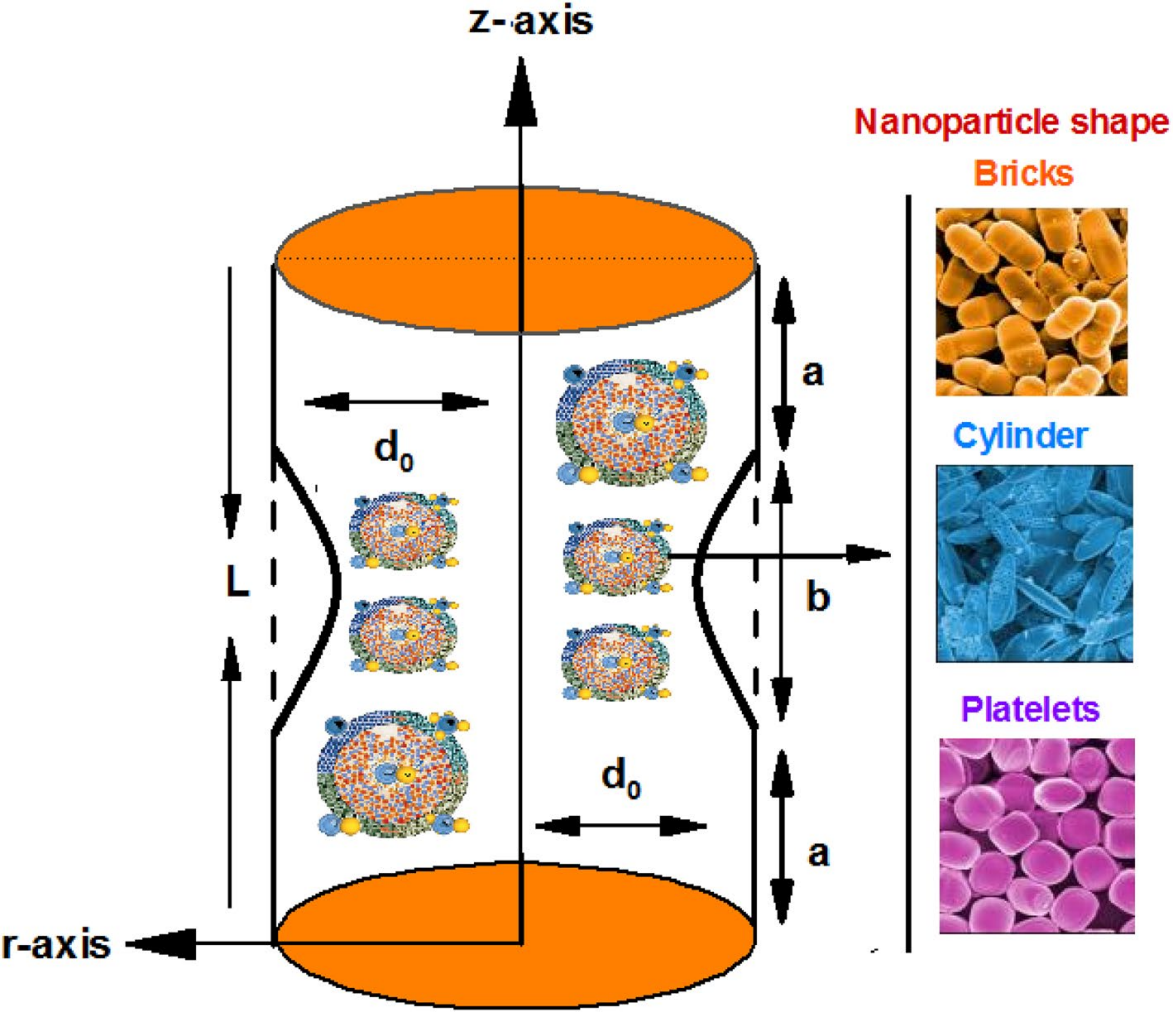
Geometry of the problem.

Iincompressible, electrically conducting nanofluid for multiple shaped nano-particles is considered through tapered stenosed arteries with length $L$. We have taken cylindrical coordinate system $(r, \theta , z)$ for current problem with $\overline{u }, \overline{v }$, $\overline{w }$ as the velocity determining components in respective directions. The equations related to incompressible and magneto-hydro-dynamic peristaltic flow of nano-fluid are expressed as in Fig. 1:

Flow equations with the relevant boundary conditions are^2,3,19,20^

1 $$ \frac{{\partial \overline{u}}}{{\partial \overline{r}}} + \frac{{\overline{u}}}{{\overline{r}}} + \frac{{\partial \overline{w}}}{{\partial \overline{z}}} = 0, $$

2 $$ \rho_{nf} \left[ {\overline{u}\frac{{\partial \overline{u}}}{{\partial \overline{r}}} + \overline{w}\frac{{\partial \overline{u}}}{{\partial \overline{r}}}} \right] = - \frac{{\partial \overline{p}}}{{\partial \overline{r}}} + \mu_{nf} \frac{\partial }{{\partial \overline{r}}}\left[ {2\frac{{\partial \overline{u}}}{{\partial \overline{r}}}} \right] + \frac{{2\mu_{nf} }}{{\overline{r}}}\left( {\frac{{\partial \overline{u}}}{{\partial \overline{r}}} - \frac{{\overline{u}}}{{\overline{r}}}} \right){ } + \mu_{nf} \frac{\partial }{{\partial \overline{z}}}\left[ {\left( {\frac{{\partial \overline{u}}}{{\partial \overline{z}}} + \frac{{\partial \overline{w}}}{{\partial \overline{r}}}} \right)} \right], $$

3 $$ \rho_{nf} \left[ {\overline{u}\frac{{\partial \overline{w}}}{{\partial \overline{r}}} + \overline{w}\frac{{\partial \overline{w}}}{{\partial \overline{z}}}} \right] = - \frac{{\partial \overline{p}}}{{\partial \overline{z}}} + \mu_{nf} \frac{\partial }{{\partial \overline{z}}}\left[ {2\frac{{\partial \overline{w}}}{{\partial \overline{z}}}} \right] + \frac{{\mu_{nf} }}{{\overline{r}}}\frac{\partial }{{\partial \overline{r}}}\left[ {\overline{r}\left( {\frac{{\partial \overline{u}}}{{\partial \overline{z}}} + \frac{{\partial \overline{w}}}{{\partial \overline{r}}}} \right)} \right]{ } + \rho_{nf} g\alpha \left( {\overline{T} - \overline{T}_{0} } \right) - \sigma B_{o}^{2} \overline{w}, $$

4 $$ \left[ {\overline{u}\frac{{\partial \overline{T}}}{{\partial \overline{r}}} + \overline{w}\frac{{\partial \overline{T}}}{{\partial \overline{z}}}} \right] = \alpha_{nf} \left[ {\frac{{\partial^{2} \overline{T}}}{{\partial \overline{r}^{2} }} + \frac{1}{{\overline{r}}}\frac{{\partial \overline{T}}}{{\partial \overline{r}}} + \frac{{\partial^{2} \overline{T}}}{{\partial \overline{z}^{2} }}} \right] + Q_{0} . $$

with the conditions^3,4^

5 $$ \begin{gathered} \frac{{\partial \overline{w}}}{{\partial \overline{r}}} = 0,{ }\frac{{\partial \overline{T}}}{{\partial \overline{r}}} = 0\;{\text{at}}\;\overline{r} = 0, \hfill \\ \overline{w} = 0,{ }\overline{T} = \overline{T}_{0} \;{\text{at }}\;\overline{r} = \overline{h}\left( {\overline{z}} \right), \hfill \\ \end{gathered} $$

where the geometry for stenosis is taken as^3,4^:

6 $$ \begin{gathered} \overline{h}\left( {\overline{z}} \right) = d\left( z \right)\left[ {1 - \eta \left( {b^{n - 1} \left( {z - a} \right) - \left( {z - a} \right)^{n} } \right)} \right], \hfill \\ a \le z \le a + b,\;d\left( z \right) = d_{0} + \xi z, \hfill \\ \end{gathered} $$

7 $$ a \le z \le a + b,\;d\left( z \right) = d_{0} + \xi z,\eta = \frac{{\delta n^{{\frac{n}{n - 1}}} }}{{d_{0} b^{n} \left( {n - 1} \right)}}, z = a + \frac{b}{{n^{{\frac{n}{n - 1}}} }}. $$

Equations discussed above contain $\overline{r }$/$\overline{z }$ as coordinates in which $\overline{z }$ considered along the center line whereas $\overline{r }$ perpendicular to it. $\overline{u }$/$\overline{v }$ show velocity elements along $\overline{r }$ and $\overline{z }$ respectively. $T$ shows local temperature field. In addition, effective density dynamic viscosity, heat capacitance are expressed as ${\rho }_{nf}$/${\mu }_{nf}$/$(\rho {c}_{p}{)}_{nf}$ respectively. ${\alpha }_{nf}$ depicts effective thermal diffusibility, and ${k}_{nf}$ stands for effective thermal conductivity of the nanofluid. Formulas for these properties are mentioned as under (see refs.^18^).

$$ \begin{gathered} \rho_{nf} = \left( {1 - \varphi } \right)\rho_{f} + \varphi \rho_{f} , \mu_{nf} = \frac{{\mu_{f} }}{{\left( {1 - \varphi } \right)^{2.5} }}, \hfill \\ \alpha_{nf} = \frac{{k_{nf} }}{{\left( {\rho c_{p} } \right)_{nf} }},\;\left( {\rho c_{p} } \right)_{nf} = \left( {1 - \varphi } \right)\left( {\rho c_{p} } \right)_{f} + \varphi \left( {\rho c_{p} } \right)_{s} . \hfill \\ \end{gathered} $$

Here $\varphi$ is the solid nanoparticle volume fraction.

8 $$ k_{nf} = k_{f} \left( {\frac{{k_{s} + \left( {m + 1} \right)k_{f} - \left( {m + 1} \right)\left( {k_{f} - k_{s} } \right)\varphi }}{{k_{s} + \left( {m + 1} \right)k_{f} + \varphi \left( {k_{f} - k_{s} } \right)}}} \right), $$

where ${k}_{s}$ and ${k}_{f}$ are the conductivities of the particle material and the base fluid. $m$ is the shape factor with values expressed through Table 1.

**Table 1:** Nanoparticles shape with their shape factor.

Nanoparticles type	Shape	Shape factor
Bricks		3.7
Cylinders		4.9
Platelets		5.7

Non-dimensional variables are considered as:

9 $$ \begin{gathered} r = \frac{{\overline{r}}}{{d_{0} }},\;z = \frac{{\overline{z}}}{b},\;\;w = \frac{{\overline{w}}}{{u_{0} }},\;u = \frac{{b\overline{u}}}{{u_{0} \delta }},\;p = \frac{{d_{0}^{2} \overline{p}}}{{u_{0} b\mu_{f} }}, h = \frac{{\overline{h}}}{{d_{0} }}, \hfill \\ {\text{Re}} = \frac{{\rho bu_{0} }}{\mu }, \theta = \frac{{\left( {\overline{T} - \overline{T}_{0} } \right)}}{{\overline{T}_{0} }}, Pr = \frac{{\mu_{f} c_{p} }}{k},P_{r} = \frac{\nu }{\alpha }, \hfill \\ \beta = \frac{{Q_{0} d_{0}^{2} }}{{\overline{T}_{0} k_{f} }}, M^{2} = \frac{{\sigma B_{0}^{2} d_{0}^{2} }}{{\mu_{f} }}, G_{r} = \frac{{g\alpha d_{1}^{2} \overline{T}_{0} }}{\nu b}. \hfill \\ \end{gathered} $$

Upon using Eqs. (6), (7) with incorporating additional conditions^4^,

10 $$ \left( {\text{i}} \right) \;\frac{{{\text{Re}} \delta^{*} n^{{\left( {\frac{1}{n - 1}} \right)}} }}{b} < < 1, $$

11 $$ \left( {{\text{ii}}} \right) \;\frac{{d_{0} n^{{\left( {\frac{1}{n - 1}} \right)}} }}{b} \sim O\left( 1 \right). $$

The dimensionless equations are obtained after considering the assumption given in Eqs. (10), (11) of mild stenosis case. For the case of mild stenosis $\left(\frac{{\delta }^{*}}{{d}_{0}}<<1\right)$, Eqs can take the form:

12 $$ \frac{\partial P}{{\partial r}} = 0, $$

13 $$ \frac{\partial P}{{\partial z}} = \left( {\frac{1}{{\left( {1 - \varphi } \right)^{2.5} }}} \right) \left( {\frac{1}{r} \frac{\partial }{\partial r}\left[ {r\left( {\frac{\partial w}{{\partial r}}} \right)} \right]} \right) - M^{2} \left( {w + 1} \right) + G_{r} \theta , $$

14 $$ 0 = \frac{1}{r}\frac{\partial }{\partial r}\left( {r\frac{\partial \theta }{{\partial r}}} \right) + \beta \left( {\frac{{k_{s} + \left( {m + 1} \right)k_{f} + \varphi \left( {k_{f} - k_{s} } \right)}}{{k_{s} + \left( {m + 1} \right)k_{f} - \left( {m + 1} \right)\left( {k_{f} - k_{s} } \right)\varphi }}} \right). $$

The corresponding boundary conditions are

15 $$ \frac{\partial w}{{\partial r}} = 0, \frac{\partial \theta }{{\partial r}} = 0{\text{ at }}r = 0,\;\;w = 0,{ }\theta = 0{\text{ at }}r = h\left( z \right), $$

which

16 $$ h\left( z \right) = \left( {1 + \xi z} \right)\left[ {1 - \eta_{1} \left( {\left( {z - \sigma } \right) - \left( {z - \sigma } \right)^{n} } \right)} \right],\;\;\sigma \le z \le \sigma + 1, $$

17 $$ \eta_{1} = \frac{{\delta n^{{\frac{n}{n - 1}}} }}{{\left( {n - 1} \right)}},{ }\delta = \frac{{\delta^{*} }}{{d_{0} }},{ }\sigma = \frac{a}{b},{ }\xi^{\prime} = \frac{\xi b}{{d_{0} }}. $$

In above equations $\xi ={\text{tan}}\phi$, $\phi$ is called tapered angle whereas $\phi >0$ is diverging tapering parameter as mentioned via^4^.

## Mathematical results

The dimensionless equations provided by Eqs. (13), (14) are solved with the relevant boundary conditions given in Eq. (15) and exact solutions are computed that are given in the following section. These non-homogeneous but linear ordinary differential equations given in Eqs. (13), (14) can be solved easily by using the DSolve command in Mathematica software. We have used the DSolve command in Mathematica software to solve these ODEs with relevant boundary conditions. Firstly, solve Eq. (14) for $\theta$ with relevant temperature boundary conditions given in Eq. (15) and then insert the exact solution of $\theta$ into momentum Eq. (13) and then solve the momentum equation with boundary conditions given in Eq. (15) to get an exact solution of momentum profile. Non-homogeneous but linear set of Eqs. (13), (14) with variable coefficients, and the mathematical software Mathematica 9 is used to analyse exact solutions using the Undetermined Coefficients Method (UCM). Boundary condition solutions can be expressed as:

18 $$ w\left( {r,z} \right) = \left( {\frac{{I_{0} \left( {M\left( {1 - \varphi } \right)^{\frac{5}{4}} r} \right) - I_{0} \left( {M\left( {1 - \varphi } \right)^{\frac{5}{4}} h} \right)}}{{I_{0} \left( {M\left( {1 - \varphi } \right)^{\frac{5}{4}} h} \right)}}} \right)\left( {\frac{dP}{{dz}}\frac{1}{{M^{2} }} + \Omega_{1} } \right), $$

19 $$ \frac{dP}{{dz}} = \frac{{\left( {1 - \varphi } \right)^{\frac{5}{4}} I_{0} \left( {M\left( {1 - \varphi } \right)^{\frac{5}{4}} h} \right)\left( {2M^{4} F - \left( {Mh} \right)^{2} } \right) - L_{1} M^{2} \left( {2Mh} \right)I_{1} \left( {M\left( {1 - \varphi } \right)^{\frac{5}{4}} h} \right)}}{{\left( {I_{1} } \right)\left( {\left( {1 - \varphi } \right)^{\frac{5}{4}} hM} \right) - h^{2} M^{2} \left( {1 - \varphi } \right)^{\frac{5}{4}} \left( {I_{0} } \right)\left( {\left( {1 - \varphi } \right)^{\frac{5}{4}} hM} \right)}}, $$

20 $$ \theta \left( {r,z} \right) = \left( {\frac{{k_{s} + \left( {m + 1} \right)k_{f} + \varphi \left( {k_{f} - k_{s} } \right)}}{{k_{s} + \left( {m + 1} \right)k_{f} - \left( {m + 1} \right)\left( {k_{f} - k_{s} } \right)\varphi }}} \right)\beta \left( {\frac{{h^{2} - r^{2} }}{4}} \right), $$

### Resistance impedance

Resistance impedance formula, using Eq. (19), takes the form:

21 $$ \overline{\lambda } = \frac{\Delta p}{F} = \left\{ {\mathop \smallint \limits_{0}^{a} \left. {R\left( z \right)} \right|_{h = 1} dz + \mathop \smallint \limits_{a}^{a + b} R\left( z \right)dz + \mathop \smallint \limits_{a + b}^{L} \left. {R\left( z \right)} \right|_{h = 1} dz} \right\}, $$

Simplification of Eq. (21) yields

22 $$ \begin{gathered} \overline{\lambda } = \left\{ {\left( {L - b} \right)\left( {\frac{{\left( {1 - \varphi } \right)^{\frac{5}{4}} I_{0} \left( {\left( {1 - \varphi } \right)^{\frac{5}{4}} M} \right)\left( {2M^{4} - \frac{{\left( M \right)^{2} }}{F}} \right) - \Omega_{1} M^{2} \left( {2M} \right)I_{1} \left( {M\left( {1 - \varphi } \right)^{\frac{5}{4}} } \right)/F}}{{I_{1} \left( {M\left( {1 - \varphi } \right)^{\frac{5}{4}} } \right) - M^{2} \left( {1 - \varphi } \right)^{\frac{5}{4}} I_{0} \left( {M\left( {1 - \varphi } \right)^{\frac{5}{4}} } \right)}}} \right)} \right. \hfill \\ \quad \quad \quad \left. { + \mathop \smallint \limits_{a}^{a + b} R\left( z \right)dz} \right\}. \hfill \\ \end{gathered} $$

### Boundary shearing stresses

The boundary shearing stress expression taken as:

23 $$ \tilde{S}_{rz} = \left. {\left[ {\left( {\frac{\partial w}{{\partial r}}} \right)} \right]} \right|_{r = h} . $$

For stenosed throat, the boundary shear at its maximum height located at $z=\frac{a}{b}+\frac{1}{{n}^{\frac{n}{n-1}}}$, are defined as:

24 $$ \left. {\tilde{\tau }_{s} = \tilde{S}_{rz} } \right|_{{\left( { h = 1 - \delta } \right)}} $$

## Validation with experimental data

The experimental study on blood flow with nanoparticles is referred as in Refs.^35–39^. These experiments also show that nanoparticles play a vital role in the targeted drug delivery for cancer therapy. Experimental results also depict an axially symmetric flow profile for blood flow regions. A maximum velocity is disclosed in the central region of arterial cross-section that declines toward boundaries. The wall shear stresses attain maximum values in the stenosis regions. An increase in wall shear stresses is noted due to stenosis section. Experimental studies also validate the enhancement of thermal conductivity by adding different kinds of nanoparticles.

## Analysis and discussion

This section is written for the analysis of stenosis shape factor n, in addition to the Grashof number (${G}_{r}$), maximum height ($\delta$) of the stenosis, the Hartmann number ($M$), the heat sink parameter ($\beta$), and the nano-particle volume percentage ($\varphi$) on diverging tapered arteries. Converging tapering happens when $\left({\varphi }_{1}\succ 0\right)$ and is indicated by the tapered angle ${\varphi }_{1}$. Cu-water (copper water) has undergone analysis. Tables 1 and 2 illustrate the physical characteristics of Cu-water shaped particles. It is evident in Table 2 that the base fluid water has a very low thermal conductivity that restricts the heat transfer analysis for present study case. Thus, copper nanoparticles are added in this base fluid to achieve a nanofluid that has a high thermal conductivity since Cu has a high thermal conductivity as shown in Table 2. Hence the higher thermal conductivity of the composed nanofluid makes it possible to analyze the heat transfer results. Thus the addition of Cu nanoparticles has a vital role to enhance the thermal conductivity of the considered system that helps in the analysis of heat transfer similar behaviour is observed in Refs.^35–39^.

**Table 2:** Thermal properties of base fluid and nanoparticles.

Physical properties	Fluid phase (water)	Cu
$\rho$ (J/kgK)	4179	385
${c}_{p}$ (kg/m^3^)	997.1	8933
*k* (W/mk)	0.613	400

### Velocity profile

**Figure 2:**
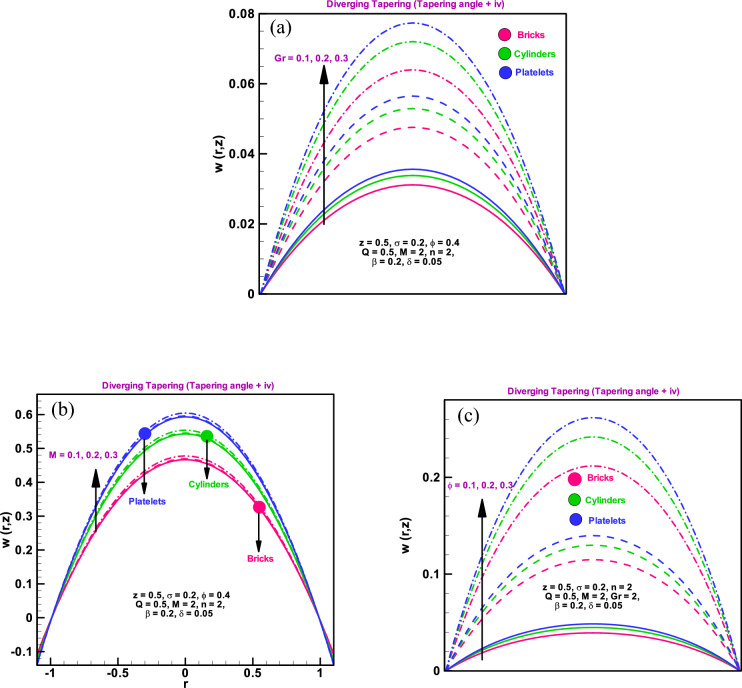
(**a**) Velocity profile for different dimensionless parameters ${G}_{r}=0.1, 0.2, 0.3$. (**b**) Velocity profile for different parameters $M=0.1, 0.2, 0.3$. (**c**) Velocity profile for different dimensionless parameters $\varphi =0.1, 0.2, 0.3.$

This subsection addresses the effect of related parameters on axial velocity through Fig. 2a–c. From these Figures, it can be seen that higher Grashof number (${G}_{ r}$), which means larger buoyant effect than viscous forces, the velocity profile for bricks/cylinders/platelets in the case of diverging tapering arteries rises rapidly. The reason behind this behaviour is the greater buoyancy forces. Because there will be more gravity as a result of the high buoyancy forces, the blood will move more quickly, which will cause the velocity field to rise, because increasing gravitational forces can influence various aspects of blood flow in arteries, impacting blood pressure, venous return, and peripheral resistance. These effects are part of the body's complex mechanisms for maintaining cardiovascular homeostasis in different postures and gravitational environments. For all the varied shaped particles, such as bricks, cylinders, and platelets, the velocity profile increases quickly when the electromagnetic force is high relative to the viscous force, as shown in Fig. 2b and similar behaviour is observed in Refs.^35–39^. High electromagnetic forces could theoretically influence the behavior of individual blood cells. For instance, red blood cells, which contain iron, might experience slight movement or alignment in the presence of strong magnetic fields. However, the physiological impact on blood flow at the macroscopic level within arteries is considered negligible. According to Fig. 2c, the velocity of brick, cylinder, and platelet particles rapidly decreases when the form of the stenosis changes. Additionally, it has been observed that as blood copper levels rise, arteries become much more relaxed, blood flow increases, and the velocity fields of all brick, cylinder, and platelet types particles gradually increase. Additionally, it is observed that for all parameters, the velocity for platelets is higher than for bricks and cylinder-shaped particles. An axially symmetric blood flow profile is evident for the considered dimensionless parameters in all velocity graphs. Blood flow attains maximum velocity in the central region of considered cross-section that declines towards the walls of stenosed artery same analysis is observed in Refs.^35–39^.

### Shear stress at boundary

**Figure 3:**
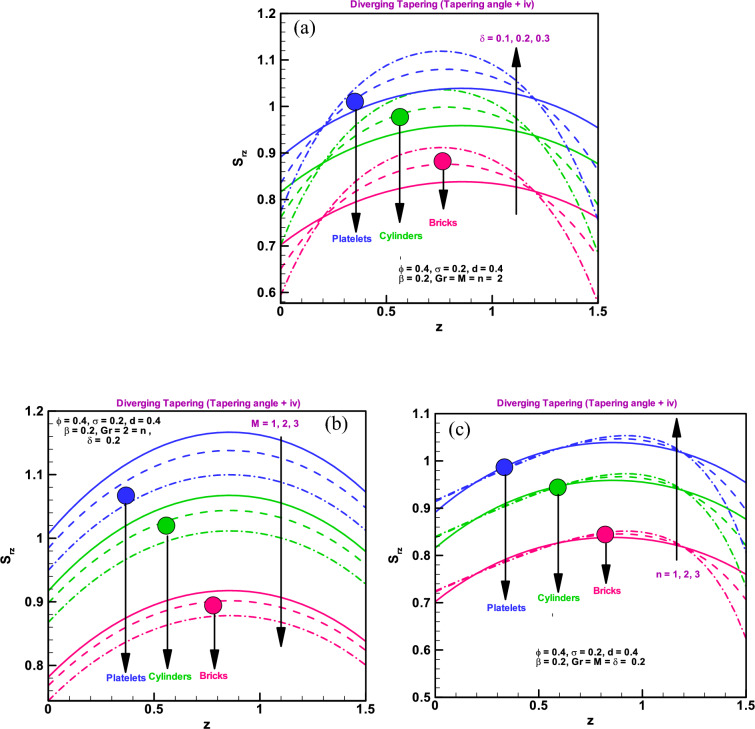
(**a**) Wall shearing stress for different dimensionless parameters (**a**) $\delta =0.1, 0.2, 0.3$. (**b**) Wall shearing stress for different dimensionless parameters $M=1, 2, 3$. (**c**): Wall shearing stress for different dimensionless parameters $n=1, 2, 3$.

Figure 3a–c illustrate how the diverging arteries affect the boundary shearing stresses, (${S}_{rz}$), for brick-, cylinder-, and platelet-type particle shapes via Cu-water nanofluid. The tapered stress yield is diverging and has a tapered angle of $({\varphi }_{1}<0$). For whole differently shaped nano-elements, such as bricks/cylinders/platelets, a rapid increase in shear stress with increasing stenosis height is observed (Fig. 3a). It is evident from Fig. 3b, shear stress decreases quickly for whole system of various shaped nano-particles, including bricks, cylinders, and platelets, when electromagnetic forces are strong compared to viscous forces. Shear stress rises when the stenosis's form changes, as shown in Fig. 3c. Additionally, shear stress gives larger values for platelet-shaped particles across the board than it does for brick- and cylinder-shaped particles. The shear stress graphs show the exact formation of stenosis at the boundary as seen in Refs.^35–39^ as well.

### Temperature profile

**Figure 4:**
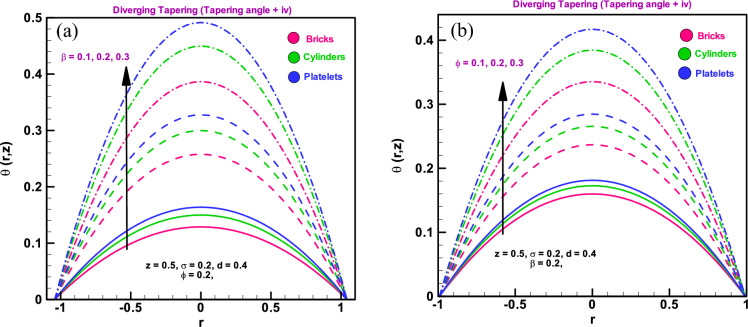
(**a**) Temperature profile for different dimensionless parameters $\left(a\right)$ $\beta =0.1, 0.2, 0.3.$ (**b**) Temperature profile for different dimensionless parameters $\varphi =0.1, 0.2, 0.3.$

The thermal analysis for various values of the heat sink parameter ($\beta$) and the volume percentage of the nano-elements (ϕ), is shown in Fig. 4a and b. It has been found that all shaped particles, including bricks, cylinders, and platelets, exhibit a rapid increase in temperature profile with an increase in the heat absorption parameter $\beta$. It has been observed that when the amount of copper in water increases, arteries become more flexible and all formed particles, including bricks, cylinders, and platelets, experience gradual temperature increases. The temperature profile has maximum value in the central region of considered arterial cross-section that declines towards the walls of the stenosed artery the similar behaviour is observed in Refs.^35–39^.

### Impedance resistance

**Figure 5:**
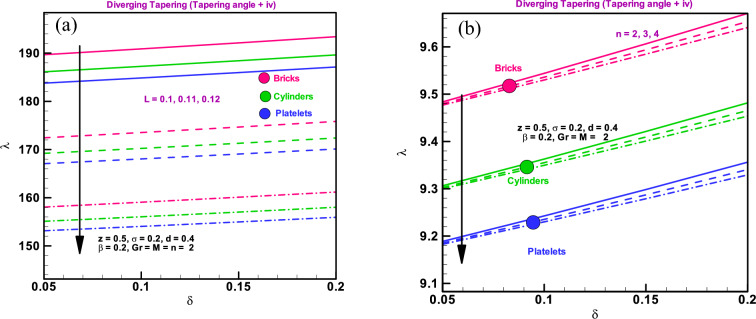
(**a**) Impedance resistance for different dimensionless parameters $L=0.1, 0.11, 0.12$. (**b**) Impedance resistance for different dimensionless parameters $n=2, 3, 4.$

As shown in Fig. 5a through b, increasing $L$ and $n$ for all shapes of particles, including bricks, cylinders, and platelets, causes the impedance resistance for diverging tapering arteries to drop. Compared to platelets and cylinders, the largest impedance resistance is seen in brick-type particles.

### Shearing stress impact on stenosed throat

**Figure 6:**
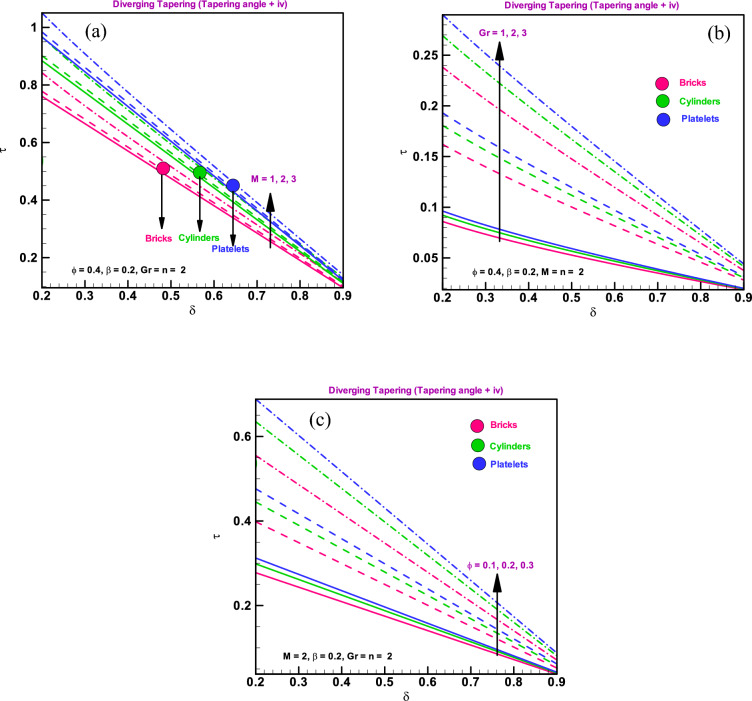
(**a**) Shear stress for stenosed throat for different dimensionless parameters $M=1, 2, 3$. (**b**) Shear stress for stenosed throat for different dimensionless parameters ${G}_{r}=1, 2, 3$. (**c**) Shear stress for stenosed throat for different parameters $\varphi =0.1, 0.2, 0.3.$

In order to show the fluctuation of the shearing stress at the stenosis throat ${\tau }_{s}$ with $\delta$, Fig. 6a–c are constructed. At the throat of the stenosis, shearing stress occurs because the arteries there are very, very small. We discovered that for all shapes of particles, including bricks, cylinders, and platelets, tiny arteries result from shearing stress at the stenosis throat when electromagnetic force is large relative to viscous force, as shown in Fig. 6a. Additionally, it is shown that shearing stress at the throat ${\tau }_{s}$ exhibits similar behaviour for Grashof number ${G}_{r}$, i.e., it increases for all shapes of particles, such as bricks, cylinders, and platelets, when buoyancy forces are higher than viscous forces (Fig. 6b). According to Fig. 6c, shearing stress, which is experienced at throat, is significant for various shapes of nano-particles, including bricks, cylinders, and platelets, for high copper rates as seen in Refs.^35–39^.

### Streamlines pattern

**Figure 7:**
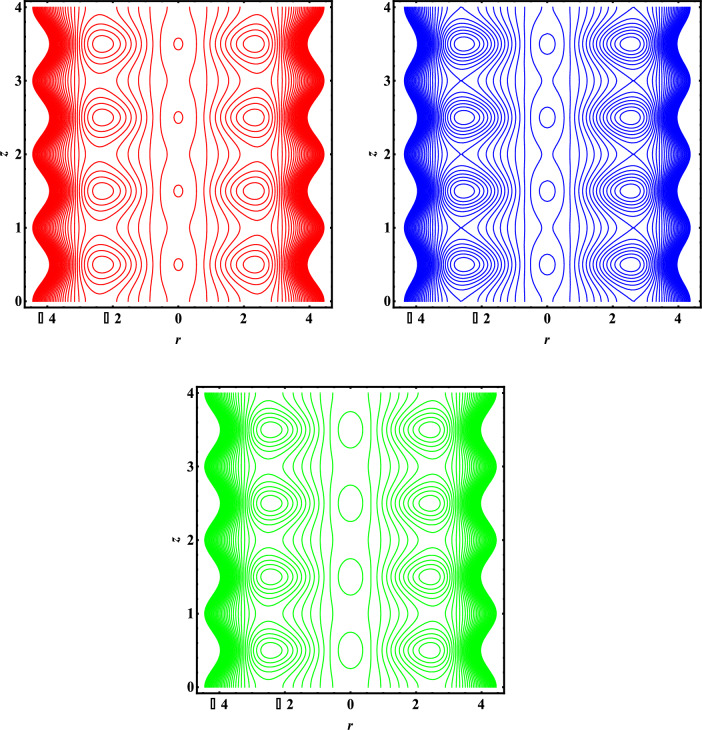
Streamlines pattern for particles with different shapes. Red. (Platelets). Blue (Cylinders). Green (Bricks).

Figure 7a–c discuss the phenomenon of trapping. When streamlines are about to be enclosed, this phenomenon happens. Figure 7a through c display the stream lines patter for various shaped particles, comprised of bricks/cylinders/platelets. As opposed to cylinder and platelets, it has been found that there are more trapped boluses for brick-like particles. However, compared to platelets and bricks, the size of the trapped bolus is larger for cylinder-type particles. Table 1. Shows the shape factor of nanoparticles and Table 2 gives the thermophysical properties of nanoparticles and base fluids. Table 3. Gives the comparison of present results with existing literature.

**Table 3:** Give the comparison of present results with the existing literature.

r	w(r,z) when ${G}_{r}=0$,$\varphi =0$	w(r,z) Ref.^2^	w(r,z) Ref.^4^, N = m = 0
− 1.0	0.00000	0.00000	0.00000
− 0.8	0.12345	0.12351	0.12325
− 0.6	0.29546	0.29563	0.29525
− 0.4	0.41236	0.41252	0.41234
− 0.2	0.51234	0.51261	0.51227
0	0.61357	0.61386	0.61379
0.2	0.51234	0.51254	0.51256
0.4	0.41236	0.41276	0.41268
0.6	0.29546	0.29556	0.29564
0.8	0.12345	0.12364	0.12353
1.0	0.00000	0.00000	0.00000

## Conclusions

The blood flow in diverging tapering stenosed arteries is examined using variously shaped nanoparticles. The use of metallic nanoparticles in various shapes with water as the base fluid in connection with stenosis has not yet been investigated. The following are the main points of the current analysis.

1. It has been found that high Grashof numbers indicate buoyancy forces that are higher than viscous forces, causing brick, cylinder, and platelet velocity profiles to climb fast in diverging tapering artery cases.
2. When electromagnetic force is strong in comparison to viscous force, the velocity profile of all the varied shaped particles, including bricks, cylinders, and platelets, rapidly increases.
3. It is seen that the velocity of brick, cylinder, and platelet particles rapidly decreases as the geometry of the stenosis changes.
4. It has also been discovered that as blood copper levels rise, arteries become much more relaxed, blood flow increases, and the velocity fields of all brick, cylinder, and platelet-type particles gradually increase.
5. It is further observed that for all parameters, the velocity for platelets is higher than for bricks and cylinder-shaped particles.
6. The stress yield is tapering and convergent at a tapered angle ϕ_1_ < 0.
7. Shear stress increases quickly for all shaped particles, including bricks, cylinders, and platelets, as the stenosis height increases.
8. For all parameters, shear stress results in large values for platelet-shaped particles compared to brick- and cylinder-shaped particles.
9. Compared to platelets and cylinders, brick-type particles exhibit the highest levels of impedance resistance.
10. The shearing stress at the stenosis throat increases when electromagnetic force is greater than viscous force, which results in smaller arteries for all shaped particles.
11. The size of the trapped bolus for platelet-type particles is bigger than that of cylinders and blocks.

## Data Availability

The datasets used and/or analysed during the current study available from the corresponding author on reasonable request.
